# The Burden of Anxiety During the COVID-19 Pandemic Among People Living with HIV (PLHIV) in Pune, India

**DOI:** 10.21203/rs.3.rs-45412/v1

**Published:** 2020-08-13

**Authors:** Ivan Marbaniang, Shashikala Sangle, Smita Nimkar, Kanta Zarekar, Sonali Salvi, Amol Chavan, Amita Gupta, Nishi Suryavanshi, Vidya Mave

**Affiliations:** Byramjee Jeejeebhoy Government Medical College - Johns Hopkins University Clinical Research Site; Byramjee Jeejeebhoy Government Medical College; Byramjee Jejeebhoy Government Medical College - Johns Hopkins University Clinical Research Site; Byramjee Jeejeebhoy Government Medical College - Johns Hopkins University Clinical Research Site; Byramjee Jeejeebhoy Government Medical College; Byramjee Jeejeebhoy Government Medical College - Johns Hopkins University Clinical Research Site; Johns Hopkins University School of Medicine; Byramjee Jeejeebhoy Government Medical College - Johns Hopkins University Clinical Research Site; Byramjee Jeejeebhoy Government Medical College - Johns Hopkins University Clinical Research Site

**Keywords:** India, COVID-19 pandemic, Poverty, GAD-7, Anxiety, Screening

## Abstract

**Introduction:**

There is a dearth of data on anxiety related to the COVID-19 pandemic from people living with HIV (PLHIV). This is a cause of concern as anxiety is associated with antiretroviral therapy (ART) nonadherence. Globally, India has the third largest population of PLHIV and third highest number of COVID-19 cases which are rapidly increasing. Therefore, it is crucial to understand the burden of anxiety and its sources among Asian Indian PLHIV during this pandemic.

**Methods:**

We used data from a telephonically delivered assessment among PLHIV engaged in care at a tertiary healthcare associated antiretroviral therapy (ART) center in Pune, India. Assessments were conducted between April 21 and May 28, 2020, one month into the government mandated lockdown. GAD-7 was used to assess for anxiety over two-preceding weeks. Significant sociodemographic and clinical differences between groups (GAD-7<10 and GAD-7≥10) were assessed using Fisher’s exact and Wilcoxson rank sum tests, for categorical and continuous variables, respectively. Thematic analysis was employed to analyze an open-ended question that asked about the most pressing cause(s) of concern.

**Results:**

Of 167 PLHIV contacted, median age was 44 years (IQR:40 – 50), 60% (n=100) were cisgender women and 81% (n=135) had a monthly family income<200 USD. Thirty-eight percent (n=64) had prior history of tuberculosis and 27% (n=45) were living with another comorbidity. A fourth (25%, n=41) had GAD-7 scores indicative of generalized anxiety. PLHIV who had fewer remaining doses of ART had significantly higher GAD-7 scores compared to those that had more doses (p=0.05). Thematic analysis indicated that concerns were both health related and unrelated, and stated temporally. Present concerns were often also projected as future concerns.

**Conclusions:**

In a group of socioeconomically disadvantaged PLHIV, a fourth were found to have anxiety, that appeared to be influenced by concerns about ART availability. Furthermore, the persistence of sources of anxiety and therefore an increase in anxiety for these PLHIV is anticipated as the pandemic worsens in India. We recommend the regular utilization of short screening tools for anxiety to monitor and triage PLHIV as an extension of current HIV-services.

## Introduction

Across the world, the COVID-19 pandemic and subsequent lockdowns authorized by governments as containment measures have had a profound impact on mental health. Studies from diverse settings have consistently reported an increase in the burden of mental health conditions during this period [1–3]. It is however recognized that the differential power structures which shape social hierarchies, could also be instrumental in making certain groups more prone to worse mental health [4]. This includes People living with HIV (PLHIV) who already have a disproportionately higher burden of mental health conditions [5]. Presently, there is a dearth of data for PLHIV, obfuscating our understanding of the pandemic’s effect on mental health among them.

From a Public Health standpoint, an important aspect of understanding mental health among PLHIV is the association it has with HIV treatment outcomes and transmission dynamics. Depression is associated with treatment failure [6, 7], lower CD4 count and risky sexual behavior [8, 9], and anxiety with disengagement from care [10, 11]. The importance of generating data on mental health among PLHIV, in the setting of the pandemic, where reported mental health is worsened, can thus be inferred.

India has the third largest population of PLHIV [12] and as of July 14, 2020 the third highest number of confirmed COVID-19 cases globally [13]. In a recently published meta-analysis of studies from India, anxiety or depression were identified as risk factors for ART nonadherence [14]. In yet another meta-analysis, that included data from India, anxiety was found to be associated with 70% higher odds of nonadherence [15]. Therefore, the pandemic through declining mental health, could have a crippling effect on India’s HIV response that functions within an overburdened health system [16], that is further being paralyzed by rapidly increasing COVID-19 cases [17] following the easing of lockdown restrictions on June 8, 2020 [18].

Taking into consideration the findings of the two aforementioned recent meta-analyses; assuming that in a crisis like the COVID-19 pandemic, anxiety precedes depression; and recognizing that the two conditions co-occur frequently [19], we sought to assess the burden of anxiety symptoms and their sources among PLHIV in Pune, India.

## Methods

### Study population and procedures

We used data from participants (≥ 18 years) enrolled in a 48-month prospective cohort study that is seeking to understand the development of non-communicable diseases among 400 PLHIV. The details of this study have been reported elsewhere [20]. Participants in the cohort are registered for care at the antiretroviral therapy (ART) center affiliated to Byramjee Jeejeebhoy Government Medical College and Sassoon General Hospitals (BJGMC – SGH), a publicly funded tertiary healthcare center in Pune, India. The ART center functions under the aegis of the National AIDS Control Organization (NACO): India’s premier HIV governmental agency, and currently caters to approximately 5000 PLHIV from lower and lower-middle socioeconomic backgrounds.

Pune, a city in western India, has consistently reported a high prevalence of HIV, compared to the national average (0.67% versus 0.31%) [21, 22]. It is also located in Maharashtra, a state that is worst affected by the pandemic, contributing to a third of all confirmed COVID-19 cases in the country [23]. The city announced a second lockdown on July 18, 2020 following a surge of COVID-19 cases [24].

In order to reschedule planned 4th year study visits, in view of the government mandated lockdown, participants were telephonically contacted by two study counsellors, on a phone number that had been previously consented upon. Contacted participants were additionally verbally consented for a single time point General Anxiety Disorder–7 (GAD-7) assessment in Marathi (the locally spoken language) which is freely available online [25], a COVID-19 symptoms screening based on the US CDC checklist [26], and history of exposure to COVID-19. The GAD-7 assesses anxiety symptoms using a series of 7 questions within a timeframe of 2 weeks.

An open-ended question followed these assessments, “In the present situation, what is/are the most important thing(s) that you are worried about?” For this analysis, we used data collected between April 21, 2020 and May 28, 2020.

The Ethics Committee of BJGMC-SGH approved this project.

### Statistical and qualitative analysis

Participants were first divided into two groups based on their GAD-7 scores. Those with GAD-7 scores < 10 were assigned to one group and those with scores ≥10 to another. The cut-off of GAD-7 ≥ 10 has been shown to be 89% sensitive and 82% specific for generalized anxiety disorder [27, 28]. The distributions of participants’ sociodemographic and clinical characteristics were described over these two groups. Wilcoxon rank sum and Fisher’s exact tests were used to evaluate significant differences for continuous and categorical variables, respectively. As sensitivity analyses, the cut-offs for GAD-7 were also specified at scores of 5, 8 and 15. A two-tailed p-value of 0.05 was used to infer statistical significance. All analysis was performed using Stata 16.0.

Responses to the open-ended question as noted by the counsellors, were first translated verbatim from Marathi into English, by a translator proficient in both languages. Thematic analysis was utilized to analyze the responses. Data was coded independently by two authors (IM and SN) employing an inductive approach. Codes and themes were identified directly from the responses. The results we present are situated within a broadly essentialist framework, where the material and experiential reality of participants was taken at face value [29]. Codes and themes were organized in NVivo 12.

## Results

Of 323 participants scheduled to be contacted, 52% (n = 167) were contactable. Three attempts on two separate days were made to contact all participants. The most common reasons for participants not being contactable were: i) participants choosing not to receive phone calls (36%, n = 56); ii) participants being out of cellular coverage area (33%, n = 51); iii) the phone number provided no longer being in use (25%, n = 39). None of those contacted refused the GAD-7 assessment, and the scale had high internal consistency for the study population (Cronbach’s alpha 0.96)

### Study population characteristics and findings from statistical analysis

Median age of the participants contacted was 44 years (IQR:40–50), most were cisgender women (60%, n = 100) and 81 % (n = 135) had a monthly household income of < 200 USD. Prior to the lockdown, a majority (57%, n = 95) had been employed in the informal sector. Thirty-eight percent (n = 64) had history of tuberculosis and 27% (n = 45) were living with another comorbid illness. A significant proportion were not aware of their latest CD4 counts (40%, n = 66) or viral loads (43%, n = 71) (Table 1). Two participants reported exposure to symptomatic SARS-CoV-2 individuals though none of the participants reported positive symptomatology.

Approximately 25% (n = 41) had GAD-7 scores ≥ 10. When dichotomized by GAD-7 scores, PLHIV with fewer median days of remaining ART appeared to have higher scores compared to those who had more days of ART (p = 0.05) (Table 1). This remained significant even when the GAD-7 cut-off was raised to 15 (p = 0.02). There were no significant differences observed for the other variables (Table 1). This remained true even when GAD-7 cut-offs were changed.

When stratified by gender, cisgender men living without a spouse appeared to have higher GAD-7 scores as compared to cisgender men living with a spouse (p = 0.02). GAD-7 scores were independent of living with a spouse for women (supplementary file). Similarly, we observed minimally significant higher GAD-7 scores among men whose monthly family income was <130 USD compared to those who had higher monthly family income (p = 0.09). However, these findings were no longer significant when the GAD-7 cut-off was changed to 5, 8 or 15, indicating significance to be a function of the cut-off used and hence unreliable.

### Findings from thematic analysis

The open-ended question was added after 38 participants had been contacted. A further 7 participants declined to answer the question, so thematic analysis was conducted on a subset of 122 participant responses.

Relative to cause(s) for concern, assessed through the open-ended question, four themes were identified. These were a) Concerns related to the immediate present; b) Concerns related to the imminent future; c) Lack of social and financial support; and d) Indifference to circumstances secondary to COVID-19. Themes a) and b), were further classified as health-related or health unrelated. Cognizant of the qualitative framework of thematic analysis, we do not quantify the exact number of participants that expressed each theme. However, themes a) and b) were expressed by approximately two-thirds; c) by roughly half and d) by a third of the participants.

#### a) Concerns associated with the immediate present

##### Health-related:

These were articulated as perceptions of increased susceptibility to COVID-19 or beliefs of being infected with COVID-19 in the absence of symptoms. These appeared to directly stem from participants’ self-awareness of HIV-resultant immunodeficiency.

“I have low CD4 counts and I am also taking medicines for tuberculosis. I am scared that I will get infected with coronavirus”

(age range: 40–50, cisgender man, GAD-7 score: 2)

“I have low immunity because of HIV, I am worried of getting COVID-19 infection. I feel that even a common cold could be coronavirus.”

(age range: 40–50 years, cisgender woman, GAD-7 score: 10)

##### Health unrelated:

Financial insecurity resulting from unemployment and a lack of savings, predominantly drove apprehensions about food security, eviction and the ability to provide for the family.

“I am a construction worker. I am at home with my two children. My wife is dead. Currently I am worried about how the house will run as there is no money and no work.”

(age range: 50–60, cisgender man, GAD-7 score:12)

“As the only earning member of my family, I am worried. My children are young. We are doing whatever it takes to get by, but because of the lockdown I am unemployed now. The house is rented. I cannot return to my village either.”

(age range: 40–50, cisgender man, GAD-7 score: 6)

“There is no food at home currently and I cannot feed my children. I am a housewife and I have no income or savings. The children used to earn by washing cars.”

(age range:50–60 years, cisgender woman, GAD-7 score: 10)

#### b) Concerns assocated with the imminent future

##### Health-related:

These were articulated as apprehensions about COVID-19 persistence continuing to endanger personal health, following reopening.

“I work as a care counsellor in the ART centre. There are no coronavirus patients at this time point, but I am worried what will happen if they visit the centre in the future?”

(age range: 40–50 years, cisgender man, GAD-7 score: 1)

“I am scared to return to get my medicines at the ART centre after the lockdown, if coronavirus does not end. Coronavirus must end.”

(age range: 20–30 years, cisgender woman, GAD-7 score: 5)

##### Health unrelated:

Fears about shortages of opportunities for gainful employment or dismissal from current employment fed into anxieties about an uncertain future that such eventualities would ensue. Such fears also often co-existed with an anticipation for “normality”.

“I am a sex worker. My business is closed and I have no clients because of the lockdown. I will die of hunger if the virus continues. I am worried all the time. If coronavirus doesn’t end then what?”

(age range: 30–40 years, cisgender woman, GAD-7 score: 5)

“I am going to lose my job because of this lockdown. I am eager to know when will COVID-19 end, when will we go back to normal life?”

(age range: 40–50 years, cisgender woman, GAD-7 score: 9)

“I stay with my mother and sold fruits for a living. Now that has closed, and I don’t know when I will be able to start again. When will COVID-19 end? When can we start normal life?”

(age range: 30–40 years, cisgender woman, GAD-7 score: 12)

#### c) Lack of social and financial support:

Isolation from family members and friends accompanied feelings of loneliness and helplessness, and the lack of financial buffers perpetuated these feelings.

“I stay alone. I used to run a beauty salon that I rented, which is now closed. I have no money to pay the owner who is asking for rent. I have no savings and no one to talk to. I have a lot of tension and I feel lonely.”

(age range: 40–50 years, cisgender woman, GAD-7 score: 21)

“I stay alone. My daughter is recently married. I worked in a company but it has closed. I have no salary and I stay in a rented house. I receive no help from my in-laws who stay in the same neighborhood.”

(age range: 40–50 years, cisgender woman, GAD-7 score: 10)

This theme was also common among migrant workers from outside or within the state.

“My family is in Bihar (a state 900 miles to the east). I want to go home, but I can’t. There is a lot of tension and I worry a lot. I have no work and no money now.”

(age range: 30–40 years, cisgender male, GAD-7 score: 21)

#### d) Indifference to circumstances secondary to COVID-19:

Some remained unperturbed by the pandemic and its control measures. However, this indifference appeared to be closely linked to a sense of security, by virtue of a profession, continuing employment or location.

“I work in the fields. There is no coronavirus there. Everything is fine.”

(age range: 40–50 years, cisgender man, GAD-7 score: 0)

“Now, I have work on the sewing machine and I am not worried at all.”

(age range 30–40 years, cisgender woman, GAD-7 score: 0)

“I do not get out of the house and I am not worried at all.”

(age range: 40–50 years, cisgender woman, GAD-7 score: 0)

## Discussion

A fourth of the participants had scores indicative of generalized anxiety disorder, which were not differential by age, gender, or socioeconomic background, underscoring the pervasiveness of anxiety symptoms in the current pandemic. Additionally, a range of health-related and health unrelated factors directly linked to the pandemic, affected participants’ perceptions, and shaped their present beliefs and future expectations.

Compared to a Hong Kong study conducted during the early phases of the pandemic among HIV-uninfected individuals, that utilized the same scale and had comparable age and gender distributions [30], our results are marginally higher. However, in comparison to estimates from the largest study to report on anxiety symptoms among Asian Indians PLHIV [31], our estimates are notably lower. We attribute this incongruency primarily to differences in scales and classifications used in both studies but acknowledge that perceptions of diminished vulnerability to COVID-19 among some, as evidenced in our qualitative findings, could also play a role. It is therefore imperative that our results are not interpreted in isolation, but in the context of the evolving pandemic in India.

As mentioned earlier, nonadherence to ART is one of many adverse effects of anxiety [14, 15]. While our study population is appreciably small, potential nonadherence in a fourth of it, could have far reaching consequences on viral suppression, HIV- transmission and antiviral resistance for the community [32]. Further, mental health services are not integrated within the Indian HIV-care delivery framework [33]. Accordingly, linkage of participants to these services falls outside the realm of HIV-programmatic capabilities. Given India’s severe shortage of trained mental health professionals [34], such linkages are not always feasible. In the current pandemic when mental health conditions are on the increase [35], linkages become even more challenging. Since anxiety, deficiency of mental health services and ART nonadherence are interdependent [11], it is not difficult to surmise the negative effect that the pandemic could have on India’s 90-90-90 goals. For our participants’ that had GAD-7 scores ≥ 10, we intend to follow-up with them and repeat the assessment after two months. In the event that they have persistently high GAD-7 scores, we will link them to a mental health professional at BJGMC-SGH.

**Figure 1:**
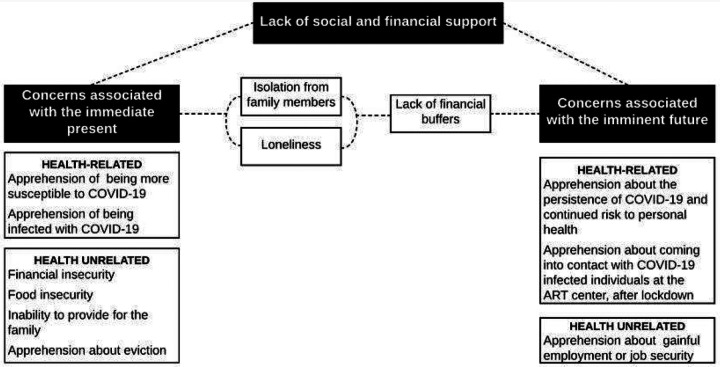
Three themes (black boxes) were identified in thematic analysis as causes of concern. As indicated by dotted lines, the themes were not always mutually exclusive. However, one theme could be a predominat cause of concern. Causes of concern also appeared to recur and their persisience implicated in the absence of mitigating measures. As an example, participants were concerned in the immediate present about not having any money to be able to provide for the family. In the absence of financial buffers such as savings, this concern was also projected into the imminent future.

We identified three themes that broadly encapsulated our participants’ cause(s) for concern, which extended across a wide range of GAD-7 scores. As exhibited in their remarks, while one theme could be a predominant cause of concern, more often than not themes were interconnected. Thus, a present cause of concern could also be a recurring future concern. It is not untenable to extrapolate from our participants’ statements that the chronicity of a particular concern is directly dependent to the rapidity with which their financial, social or apprehensions about personal health are addressed. We have represented this as a conceptual framework in Fig. 1. Although the themes we identified may not seem specific to PLHIV, they must first be contextualized to our participants existing socioeconomic backgrounds and the manner in which the pandemic will potentially affect their vertical social mobility. Secondly, they need to be understood from the aspect of how low socioeconomic status and restriction in social mobility will in turn affect HIV treatment outcomes.

More than half of our participants have a) less than the 2019–2020 estimated monthly per-capita income for India [36]; b) less than 10 years of education, and c) were employed in the informal sector or didn’t have employment prior to the lockdown. The response of the Indian government to address the financial crisis being faced by the poor as a consequence of the lockdown has been in the form of two programs, namely the Pradhan Mantri Garib Kalyan Yojana (PMGKY) and the second tranche of the Atmanirbhar Bharat [37]. These stimulus programs have been criticized as being lower than those offered by other governments [37, 38]. Analysis of the PMGKY also indicates that it has not mobilized additional funding but reallocated funding across existing budgets or allowed individuals to make advance withdrawals, raising concerns about the utility of these measures for the poor in the long run [37, 39]. Furthermore, the World Bank estimates that the COVID-19 pandemic could push a substantial section of individuals in socioeconomic positions similar to our participants into extreme poverty [40]. As the association between socioeconomic deprivation and poor mental health is well-established [41], the worsening of mental health for most of our study population is foreseeable. This would in turn affect HIV-treatment outcomes (through reduced adherence, increased antiviral resistance, etc.) for a group of disadvantaged individuals within an already vulnerable population.

Interestingly, while none of the participants expressed concern about the remaining doses of ART directly, we found PLHIV with fewer remaining doses to have significantly higher GAD-7 scores compared to those with more doses. Our finding suggests that though the concern about ART availability may not be at the forefront of our participants’ concerns, it could be instrumental in affecting anxiety levels among PLHIV. The deputy director general of NACO has assured that contrary to findings from a recent survey done by the World Health Organization (WHO) which showed several countries to be at risk for ART stock-out [42], India will not face such a crisis [43]. This is encouraging news for Asian Indian PLHIV, which will probably go a long way to allay fears about ART availability. However, Indian policy makers need to consider if access to ART is as uncompromised as ART stocks in this pandemic.

There are a few limitations to our findings. Firstly, our results cannot be extrapolated to all PLHIV in Pune, based on our small sample size. However, since the socioeconomic backgrounds of PLHIV registered for care at the ART center are largely homogenous, the reported prevalence of anxiety symptoms and consequences secondary to these anxiety levels could be generalizable to them. Secondly, as we do not have GAD-7 scores prior to the lockdown, we cannot conclude with absolute certainty that the present levels we observed are entirely attributable to the pandemic. But given that a) our participant’s expressed concerns were almost exclusively related to the pandemic; b) the GAD-7 assessment timeframe being two preceding weeks and; c) our study being carried out one month into the lockdown in India, we ascribe our observed anxiety levels for the most part to the pandemic. It is also difficult to determine whether we have underestimated or overestimated the prevalence of anxiety symptoms even within our cohort, especially given the high non-response rate. Participants with higher levels of anxiety undoubtedly could choose not to receive our calls more, but it is equally plausible that those who responded were more anxious. We also used only a single question to conduct a thematic analysis, which limits a more nuanced understanding of the issue at hand. However, using thematic analysis as a guiding framework allowed us to more concretely consolidate the wealth of information provided by our participants into definitive themes. Lastly, although we did not observe differences in GAD-7 scores by comorbidity or prior tuberculosis status, we are unable to comment on how mental health in such individuals will change over time given their higher risk for COVID-19 infection [44, 45] and what that will mean in terms of disengagement from care or HIV treatment outcomes for them. However, we are instituting longitudinal follow-ups for all our study participants and we will better understand these associations by the end of 2020.

## Conclusion

Despite our limitations, our findings provide important insights into the burden and sources of anxiety symptoms in a small group of Asian Indian PLHIV. To our knowledge, these findings are the first to be reported from India during this pandemic for PLHIV. Our findings also come with the sobering implication that the COVID-19 pandemic will have devastating effects on the mental health of Asian Indian PLHIV and downstream HIV- related treatment outcomes, especially as the pandemic continues to grow in India. This is more likely to happen for PLHIV who are socioeconomically disenfranchised. While sweeping financial assistance, along with extensive social and health support mechanisms would indeed be a panacea for COVID-related anxiety symptoms for PLHIV, we do not believe that these are practicable. Instead, we recommend that whenever possible HIV care providers make regular use of short screening tools available, to identify and prioritize PLHIV at risk for anxiety and other mental health conditions. This strategy will not redress the deleterious effects of the pandemic on HIV care, but at the minimum reduce their impact.

## Supplementary Material

Supplement

## Figures and Tables

**Table 1:** Distribution of GAD-7 scores of by sociodemographic and clinical characteristics among PLHIV contacted

	Total N (%)	GAD-7 ≥ 10 n (%)	GAD-7 < 10 n (%)	p-value
N (%)	**167**	**41 (24.6)**	**126 (75.4)**	-
Median age in years (IQR)	44 (40–50)	43 (40–49)	45 (40–50)	0.8
Gender				
Cisgender men	66 (39.5)	17 (41.5)	49 (38.9)	0.3
Cisgender women	100 (59.9)	23 (56.1)	77 (61.1)	
Transgender woman	1 (0.6)	1 (2.4)	0	
Monthly household income (USD)				
<65	35 (20.9)	11 (26.8)	24 (19.0)	0.6
65–130	64 (38.3)	14 (34.1)	50 (39.6)	
131–199	36 (21.6)	10 (24.4)	26 (20.6)	
≥ 200	32 (19.2)	6 (14.6)	26 (20.6)	
Education				
No formal education	22 (13.2)	4 (9.8)	18 (14.3)	0.4
≤ 9 years	74 (44.3)	22 (53.7)	52 (41.3)	
>9 years	71 (42.5)	15 (36.6)	56 (44.4)	
Employment prior to lockdown^†^				
Unemployed	33 (19.8)	9 (21.9)	24 (19.0)	0.5
Informal sector	95 (56.9)	25 (61.0)	70 (55.6)	
Salaried	39 (23.3)	7 (17.1)	32 (25.4)	
Living with a spouse^‡^				
Yes	83 (49.7)	19 (46.3)	64 (50.8)	0.7
No	84 (50.3)	22 (53.7)	62 (49.2)	
Median duration on ART in years (IQR)	9.8 (6.5–12.9)	9.5 (6.7–11.8)	9.9 (6.4–13.1)	0.4
Latest CD4 counts (cells/mm^3^)				
<500	44 (26.4)	10 (24.4)	34 (26.9)	0.8
≥ 500	57 (34.1)	13 (31.7)	44 (34.9)	
Do not know	66 (39.5)	18 (43.9)	48 (38.1)	
Latest viral load				
Undetectable (< 50 copies/mL)	87 (52.1)	16 (39.0)	71 (56.3)	0.1
≥ 50 copies/mL	9 (5.4)	3 (7.3)	6 (4.8)	
Do not know	71 (42.5)	22 (53.7)	49 (38.9)	
Prior history of tuberculosis				
Yes	64 (38.3)	19 (46.3)	45 (35.7)	0.3
No	103 (61.7)	22 (53.7)	81 (64.3)	
Living with another comorbidity^§^				
Yes	45 (27.0)	12 (29.3)	33 (26.2)	0.7
No	122 (73.0)	29 (70.7)	93 (73.8)	
Median days of remaining ART (IQR)	60 (28–76)	32 (17–60)	60 (30–79)	**0.05**
Discontinued HAART during the lockdown	5 (3.3)	-	-	-

ART – Antiretroviral Therapy

Median GAD-7 score for the study population was 3 (IQR: 0–9), range 0–21

† Informal sector employment for women mainly included working as house maids or domestic help (89%), for men this was mainly as daily wage laborers (92%)

‡ Living with a spouse: No includes PLHIV who are single, widowed, separated or divorced

§ Comorbidity includes having any of the following: COPD, asthma, CVD, hypertension, diabetes, renal disease, cancer.

## Abbreviations

PLHIV: People living with HIV
ART: Antiretroviral therapy
BJGMC - SGH: Byramjee Jeejeebhoy Government Medical College and Sassoon General Hospitals
NACO: National AIDS Control Organization
GAD-7: General Anxiety Disorder–7
PMGKY: Pradhan Mantri Garib Kalyan Yojana
