# Supplementary material for: The Burden of Anxiety During the COVID-19 Pandemic Among People Living with HIV (PLHIV) in Pune, India
Source: Res Sq. 2020 Aug 13:rs.3.rs-45412. Preprint. [Version 1] doi: 10.21203/rs.3.rs-45412/v1 (PMC7430601; doi:10.21203/rs.3.rs-45412/v1)
Supplement: Supplementary file 1 [file supplementarytable.docx]

**Crosstabulation between GAD-7 scores and income when stratified by gender**

**For cisgender men**

|  | Income <130 USD | Income ≥ 130 USD |
| --- | --- | --- |
| GAD-7<10 | 44.9 % (n =22) | 55.1% (n=27) |
| GAD-7≥10 | 70.6% (n=12) | 29.4% (n=4) |

Fisher’s exact p-value = 0.093

**For cisgender women**

|  | Income <130 USD | Income ≥ 130 USD |
| --- | --- | --- |
| GAD-7<10 | 67.5% (n=52) | 32.5% (n=25) |
| GAD-7≥10 | 52.2% (n=12) | 47.8% (n=11) |

Fisher’s exact p-value = 0.218

**Crosstabulation between GAD-7 scores and living with a spouse when stratified by gender**

**For cisgender men**

|  | Not living with a spouse | Living with a spouse |
| --- | --- | --- |
| GAD-7<10 | 16.3% (n=8) | 83.7% (n=41) |
| GAD-7≥10 | 47.1% (n=8) | 52.9% (n=9) |

Fisher’s exact p-value= 0.020

**For cisgender women**

|  | Not living with a spouse | Living with a spouse |
| --- | --- | --- |
| GAD-7<10 | 70.1% (n=54) | 29.9% (n=23) |
| GAD-7≥10 | 60.9% (n=14) | 39.1% (n=9) |

Fisher’s exact p-value= 0.450
